# Modeling of Steam Distillation Mechanism during Steam Injection Process Using Artificial Intelligence

**DOI:** 10.1155/2014/246589

**Published:** 2014-04-28

**Authors:** Amin Daryasafar, Arash Ahadi, Riyaz Kharrat

**Affiliations:** Petroleum Department, Petroleum University of Technology, P.O. Box 6198144471, Ahwaz, Iran

## Abstract

Steam distillation as one of the important mechanisms has a great role in oil recovery in thermal methods and so it is important to simulate this process experimentally and theoretically. In this work, the simulation of steam distillation is performed on sixteen sets of crude oil data found in the literature. Artificial intelligence (AI) tools such as artificial neural network (ANN) and also adaptive neurofuzzy interference system (ANFIS) are used in this study as effective methods to simulate the distillate recoveries of these sets of data. Thirteen sets of data were used to train the models and three sets were used to test the models. The developed models are highly compatible with respect to input oil properties and can predict the distillate yield with minimum entry. For showing the performance of the proposed models, simulation of steam distillation is also done using modified Peng-Robinson equation of state. Comparison between the calculated distillates by ANFIS and neural network models and also equation of state-based method indicates that the errors of the ANFIS model for training data and test data sets are lower than those of other methods.

## 1. Introduction

One of the most successful methods for heavy oil production is steam flooding and while steam has been injected into reservoirs almost as long, the mechanisms of this process are much less understood. Several experimental studies are performed for studying the effect of different mechanisms such as viscosity reduction, wettability alteration, and steam distillation/vaporization during this method of recovery. Among all these mechanisms, steam distillation mechanism is the main difference between steam and other thermal methods.

Steam distillation process happened when light fractions of crude oil are separated by injecting the steam into the crude oil. Observation of the produced vapors of matured steam floods proves the fact that steam can carry a large amount of light hydrocarbons in the steam distillation process. Several papers have reported the effects of steam distillation on oil recovery observed in laboratory steam displacement tests. Farouq Ali [1] estimated that 5 to 10% of the heavy oil recovery and as much as 60% of the light-oil recovery may be attributed to steam distillation mechanism. Willman et al. [2] demonstrated that steam flooding produces significantly greater oil recovery than that in flooding with hot water at the same temperature. Mainly, this is due to steam distillation. Wu and Fulton [3] reported that oil in the steam plateau of an in situ combustion process is removed mainly by steam distillation. Johnson et al. [4] showed that the oil vaporization recovery by steam ranges from 54.7 to 94.0% of immobile oil volume.

Several methods have been presented for simulating steam distillation mechanism in steam injection process. Sukkar [5] used the relative velocities of steam, the steam front, and also the rates at which hydrocarbon components were distilled to estimate the amount of oil distilled during steam flooding. Holland and Welch [6] developed a model for calculating steam distillation yield at saturated steam temperatures, where the solubility of hydrocarbon and water is negligible. Duerksen and Hsueh [7] proposed correlations for the prediction of steam distillation yield with different crude oil properties and operating conditions. They also showed that the distillation recovery correlates well with American Petroleum Institute (API) gravity and wax content. Northrop and Venkatesan [8] presented an analytical multicomponent model to predict steam distillation yield and showed that the distillation yield increases as the temperature increases. Van Winkle [9] proposed a method to predict the amount of steam required for distillation of a specific amount of a volatile material based on Raoult's and Dalton's laws.

The mentioned studies may face considerable errors when they are applied to crude oil samples and some of them require experimental data such as oil characterization data, so we need to propose a model for prediction of steam distillation yield with minimum entry data.

The complexity of steam distillation mechanism leads us to use artificial intelligence such as artificial neural network (ANN) and adaptive neurofuzzy interference system (ANFIS) for simulation of steam distillation process. In this paper we use ANN and also ANFIS to propose a practical model for predicting the steam distillation recovery as accurate as possible by choosing the best model based on laboratory data. This model can be applied to predict the steam distillation yield of crude oils with new properties (Table 2).

## 2. Description of Method

### 2.1. Artificial Neural Network (ANN)

A neural network is structured by multiple connection units arranged in layers which indicate the weights between neurons that are learned under an optimization criterion. ANNs provide a nonlinear mapping between inputs and outputs by its intrinsic ability [11]. The success in obtaining a reliable and robust network depends on the correct data preprocessing, correct architecture selection, and correct network training choice strongly [12]. Artificial neural networks have been developed for a wide variety of problems such as classification, function approximation, and prediction. Multilayer feedforward networks are the most commonly used for the function approximation. Feedforward networks consist of groups of interconnected neurons arranged in layers corresponding to input, hidden, and output layers. Once the input layer neurons are clamped to their values, the evolving starts layer by layer and the neurons determine their output and this is the reason that these networks are called feedforward. The dependence of output values on input values is quite complex and includes all synaptic weights and thresholds. Usually this dependence does not have a meaningful analytic expression. These types of network can approximate most types of nonlinear functions, irrespective of how much they are complex.

The network is trained by performing optimization of weights for each node interconnection and bias terms, until the obtained values of output become as close as possible to the actual outputs.

The type of artificial neural network used in this study was Multilayer feedforward network. We need enough experimental data for training the network. Sixteen experimental data sets were used for simulation of steam distillation in this study and these data sets are obtained from literature [10]. Thirteen crude oil data sets were used as training data and the data sets related to Shiells Canyon, Teapot Dome, and Rock Creek oil fields were considered as test data. The inputs of this network are American Petroleum Institute (API) gravity, kinematic viscosity at 37.8°C, characterization factor, and steam distillation factor, while the output is distillate recovery. Steam distillation factor is the ratio of the volumetric amount of steam injected based on cold water equivalent and the volume of initial oil. Distillate recovery is the volumetric amount of hydrocarbon distilled over initial oil volume. The volumes are calculated at standard conditions. The characterization factor and API are defined as

(1) $$\begin{array}{c} \text{characterization}\;\text{factor}=\frac{\text{average}\;\text{boiling}\;\text{point}}{\text{specific}\;\text{gravity}},\\ \text{API}=\frac{141.5}{\text{specific}\;\text{gravity}}-131.5. \end{array}$$

Levenberg-Marquardt back propagation algorithm was used for training the network [13] and the number of neurons in hidden layers was chosen according to the minimum root mean square error (RMSE) by trial and error:

(2) $$\begin{array}{c} \text{RMSE}=\frac{\mathrm{norm}\left(\text{simulated}\;\text{result}-\text{experimental}\;\text{result}\right)}{\text{sqrt}\left(\text{length}\left(\text{simulated}\;\text{result}\right)\right)\;}. \end{array}$$

Table 1 shows the results of trial and error calculations used in this study for determining the number of neurons in the hidden layer. Several networks were trained and finally a network with one hidden layer with twenty tangent sigmoid neurons was selected as the most suitable network. The neurons in the output layer have linear transfer functions. The tangent sigmoid function is defined as follows:

(3) $$\begin{array}{c} \text{tansig}\left(n\right)=\frac{2}{1+e^{-2n}}-1. \end{array}$$

### 2.2. Adaptive Neurofuzzy Inference System (ANFIS)

Adaptive neurofuzzy inference system (ANFIS) is a kind of neural network that is based on fuzzy inference system [14]. Since it integrates both neural networks and fuzzy logic principles, it has potential to capture the benefits of both in a single framework. Generally, two objectives are followed using ANFIS: integrating the best features of fuzzy systems and neural networks and their applicability to synthesize. ANFIS combines the fuzzy logic, if-then rules, and the accuracy and learning power of neural networks to make them a hybrid intelligent system. ANFIS has the ability to solve nonlinear problems. For specifying the relationship between input and output to determine the optimized distribution of membership functions, two learning methods are generally used in ANFIS. These learning methods are back propagation and hybrid. The hybrid system is a combination of propagation and least squares method [15]. In the backward pass, the error is sent back through the network in a similar manner to back propagation [16]. Hybrid systems have been used by researchers for modeling and predictions in various engineering systems. When generating a FIS using ANFIS, selecting proper parameters is very important, including the number of MF for each individual antecedent variable and also selecting proper parameters for the learning and refining process. Parameter selection and their impact on the ANFIS have been addressed in the literature [17–19].

For simulating steam distillation process another model is proposed using ANFIS. For this purpose a structure with four inputs with three *π*-shaped built-in membership functions was considered. FIS generation was done by grid partitioning. Grid partition divides the data space into rectangular subspaces using axis-paralleled partition. Π-shaped built-in membership function is given by

(4) $$\begin{array}{c} f\left(y;m,n,o,p\right)=\{\begin{array}{cc} 0 & y\leq m\\ 2{\left(\frac{y-m}{n-m}\right)}^{2} & m\leq y\leq \frac{m+n}{2}\\ 1-2{\left(\frac{y-n}{n-m}\right)}^{2} & \frac{m+n}{2}\leq y\leq n\\ 1 & n\leq y\leq o\\ 1-2{\left(\frac{y-o}{p-o}\right)}^{2} & o\leq y\leq \frac{o+p}{2}\\ 2{\left(\frac{y-p}{p-o}\right)}^{2} & \frac{o+p}{2}\leq y\leq p\\ 0 & y\geq p. \end{array} \end{array}$$

**Figure 1:**
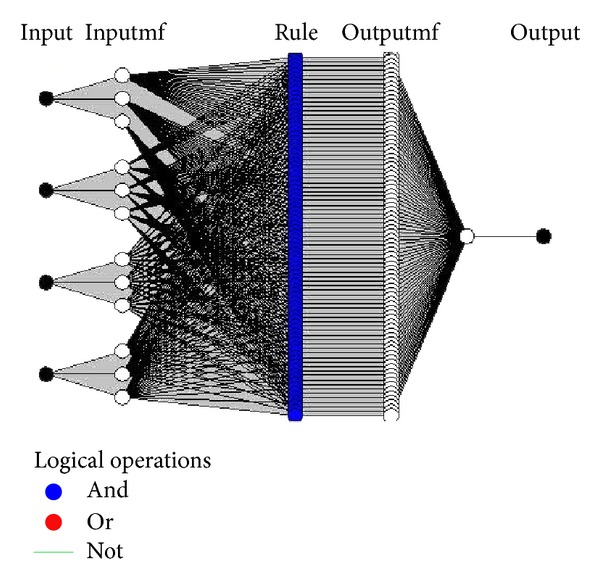
The structure of ANFIS model for steam distillation recovery estimation.

The parameters *m* and *p* locate the “feet” of the curve, while *n* and *o* locate its “shoulders.” We utilized a* hybrid* method [20] which is a combination of gradient method and least squares estimate (LSE) for training the system. The inputs of the system are American Petroleum Institute (API) gravity, kinematic viscosity at 37.8°C, characterization factor, and steam distillation factor, while the output is distillate recovery. Again, thirteen crude oil data sets were used as training data and data sets related to Shiells Canyon, Teapot Dome, and Rock Creek oil fields were considered as test data. Schematic of the proposed ANFIS structure is shown in Figure 1.

### 2.3. Equation of States Method

The first step in simulation of the steam distillation process by EOS method is to evaluate the oil characterization. This task is performed by determining data such as characterization factor, average molecular weight, viscosity, API, and distillation test data. For determining the distribution of components in liquid and vapor phases, flash calculation must be performed. It must be noticed that several equations of states must be performed and then the best EOS will be chosen as the optimum equation for simulation of this process. For this purpose, several equations of states were tested in EOS method and according to the results the modified Peng-Robinson [21] equation of state seems to generate better results [22].

In this paper, for better comparison between the proposed models and other methods, we used modified Peng-Robinson equation of state to simulate the steam distillation mechanism in steam flooding process. For this purpose, the multistage adiabatic flash calculation was performed. In this process, oil comes into contact with fresh steam in each stage, and as equilibrium condition is reached, the vapor phase which includes light fractions of oil and steam leaves the stage and the remaining oil enters the next stage.

The equation of state for mixtures proposed by Peng and Robinson [23] is as follow:

(5) P=RTV−Bm−AmV(V+Bm)+Bm(V−Bm),Bm=∑i=1Nxibi,Am=∑i=1N ∑j=1Nxixjaij0(1−kij),aij0=αiαjaiaj.

Mathias and Copeman [21] developed a density-dependent local composition (DDLC) model for the Peng-Robinson equation of state. Since the model was too expensive for computer calculation, they formulated the following truncated model:

(6) Am=∑i=1N ∑j=1Nxixjaij0(1−kij) −12BmRT2ln⁡[Z+B(1−2)Z+B(1+2)] ×∑ixiai2[∑ixjdij2−(∑jxjdij)2  ],kij=kji, dij≠dji.

## 3. Results and Discussion

The system was trained several times to achieve the best correlation between the simulated data and experimental data according to the value of mean square error (MSE), both for artificial neural network and ANFIS.

**Figure 2:**
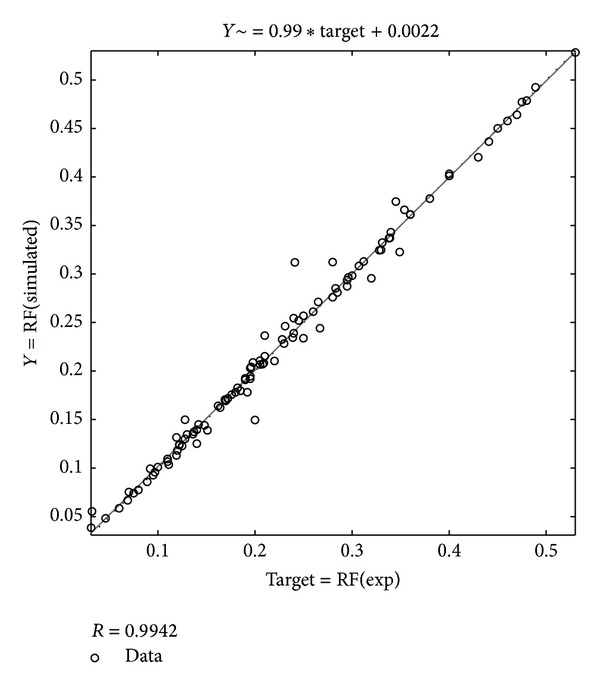
The relation between ANN predictions and actual experimental data.

In Figure 2, the best linear fit between the simulated and experimental data is illustrated with correlation factor of 0.9942 which indicates a very good correlation. These results are obtained using ANN method.

**Figure 3:**
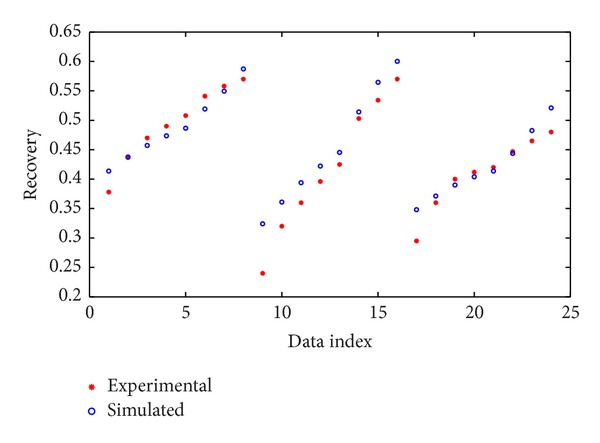
Performance of ANN for test data.

After training the network using ANN method, the network was performed on the test data and the simulation results versus experimental test data are shown in Figure 3.

**Figure 4:**
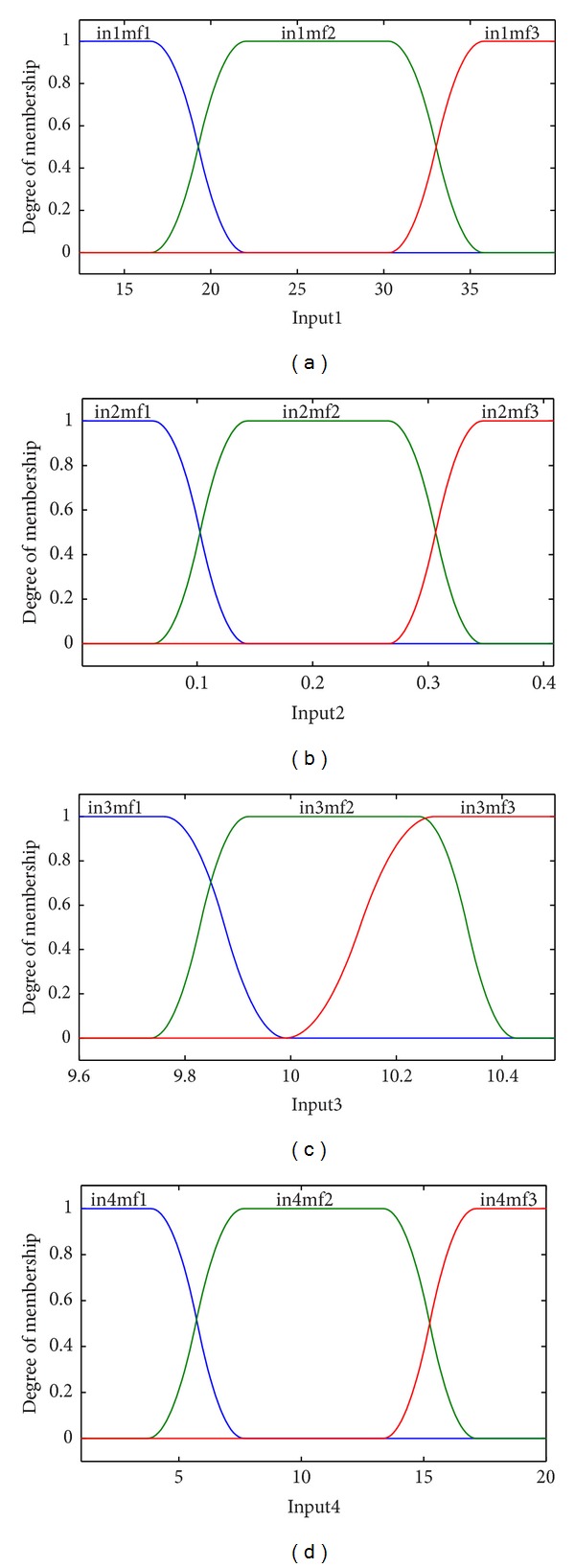
The generalized *π*-shaped membership functions of four input variables.

**Figure 5:**
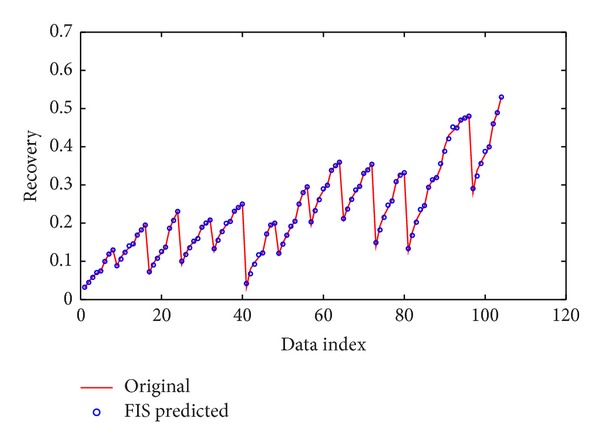
Performance of ANFIS for training data.

An ANFIS model was designed with four inputs (American Petroleum Institute (API) gravity, viscosity at 37.8°C, characterization factor, and steam distillation factor), each with three *π*-shaped built-in membership functions, and one output (distillate yield), Figure 4. We utilized a hybrid method for training the system. The results of the training data for simulated and experimental data are shown in Figure 5 which illustrates a very good correlation.

**Figure 6:**
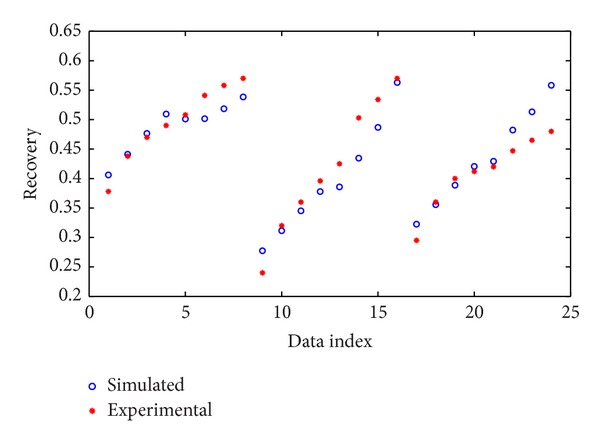
Performance of ANFIS for test data.

For validation of the proposed model by ANFIS, after training the system, it was performed on the test data and its result is illustrated in Figure 6.

In this study we also used modified Peng-Robinson equation of state, which gives the best results than those of others, to simulate the steam distillation process. Vafaei [22] found that the modified Peng-Robinson seems to generate better results and used this method to estimate the distillate yield. For validation of their estimation and then for better comparison between the results obtained by different methods and models, we again used this kind of EOS to calculate the distillate recovery. The results are given in Table 3.

The performance index used for evaluating the models is based on the present of average relative deviation (ARE) as

(7) $$\begin{array}{c} \text{ARE}=\frac{\sum_{1}^{n}\left|{\left(V_{o}/V_{oi}\right)}_{\text{sim}}-{\left(V_{o}/V_{oi}\right)}_{\exp}\right|/{\left(V_{o}/V_{oi}\right)}_{\exp}}{n}. \end{array}$$

Table 4 shows the comparison between the results obtained by different methods which were considered in this paper according to the obtained average relative error for both training and test data.

We must conclude that Vafaei [24] proposed a multilayer perceptron model for simulation of steam distillation process and used these sets of data for modeling this process but he chose White Castle, Toborg, and Teapot Dome oil fields data as test data and the remaining sets of data as training data and their model obtained ARE of 7.47% and 11.19% for training data and test data, respectively, but in this study we could achieve the less ARE by changing the conditions of each system and also choosing different sets of data for training and testing.

Comparison of the results shown in Table 4 proves that the proposed model by ANFIS gives better results for both training and test data and also using artificial intelligence can give better results with minimum entry without needing oil characterization.

## 4. Conclusion

In this paper, we proposed a model which can predict the distillate yield in distillation process accurately using ANFIS and ANN that are two important subbranches of artificial intelligence (AI) tools. ANN is one of the effective tools for function approximation but it has some problems that ANFIS can solve; for example, for reaching the best network we should run the system more times and in this study we trained the network many times to reach the best model and this is a time consuming process but ANFIS removes this problem by fuzzification process.

In this study, we utilized a FIS structure with four inputs, each with three *π*-shaped built-in membership functions, and hybrid method for training the system. Thirteen sets of data were used as training data and three sets of data as test data. The input data are the steam distillation factor, viscosity, API, and characterization factor of the oil. The obtained results by this method were compared with a multilayer feed forward neural network and also an EOS-based method. The comparison between the designed ANFIS and other two methods, that is, ANN method and EOS-based method, indicates that the accuracy of the proposed ANFIS model for both training and test data is better than that of other methods. Also both artificial intelligence models give better results than the proposed MLP model by Vafaei et al. [24].

## Figures and Tables

**Table 1:** Trial and error calculations for selecting the most suitable ANN.

Number of neurons in the hidden layer	Training data (RMSE)	Test data (RMSE)
5	0.0122	0.0316
7	0.0126	0.03
9	0.0125	0.0302
11	0.0121	0.0314
15	0.0115	0.0312
18	0.0118	0.0311
20	0.0116	0.0297
21	0.0119	0.0298
23	0.0129	0.0317
24	0.0141	0.0319
25	0.0132	0.0312

**Table 2:** Crude oil properties and experimental results of steam distillation recovery for different oil fields^a^.

Number	Field	Experimental results of steam distillation yields for *V*_*w*_/*V*_*oi*_								Crude oil properties		
		1	2	3	4	5	10	15	20	API	Kinematic viscosity at 37.8°C	Characterization factor
1	South Belridge	0.031	0.046	0.06	0.069	0.075	0.1	0.119	0.13	12.4	0.4085	9.7
2	Winkleman Dome	0.089	0.111	0.125	0.136	0.142	0.17	0.182	0.195	14.9	0.0488	9.6
3	White Castle	0.07	0.095	0.11	0.122	0.137	0.185	0.21	0.23	16	0.0308	9.7
4	Edison	0.092	0.12	0.14	0.151	0.164	0.19	0.198	0.209	16.1	0.0397	9.7
5	Red Bank	0.128	0.162	0.18	0.195	0.205	0.231	0.241	0.25	17.1	0.03	9.9
6	Slocum	0.032	0.08	0.097	0.11	0.122	0.172	0.195	0.2	18.8	0.0395	10.0
7	Hidden Dome	0.119	0.148	0.169	0.19	0.205	0.25	0.28	0.295	20.7	0.0086	10.1
8	Toborg	0.196	0.239	0.267	0.285	0.3	0.339	0.349	0.36	22.2	0.0036	10.1
9	Brea	0.21	0.24	0.265	0.283	0.296	0.33	0.34	0.354	23.5	0.0039	10.0
10	Shannon	0.14	0.192	0.22	0.24	0.26	0.307	0.328	0.331	24.7	0.0032	10.2
11	Robinson	0.128	0.176	0.208	0.228	0.245	0.295	0.312	0.32	26	0.0029	10.3
12	El Dorado	0.345	0.4	0.43	0.441	0.45	0.47	0.475	0.48	32.5	0.0005	10.1
13	Shiells Canyon	0.378	0.438	0.47	0.49	0.508	0.541	0.558	0.57	33	0.0006	10.2
14	Teapot Dome	0.24	0.32	0.36	0.396	0.425	0.503	0.534	0.57	34.5	0.0006	10.4
15	Rock Creek	0.295	0.36	0.4	0.412	0.42	0.447	0.465	0.48	38.2	0.0005	10.4
16	Plum Bush	0.28	0.338	0.36	0.38	0.4	0.46	0.489	0.53	39.9	0.0006	10.5

^a^Wu and Elder, 1983 [10].

**Table 3:** Average relative error between simulated results by EOS and experimental data.

Field	ARE%
South Belridge	19.77
Winkleman Dome	19.87
White Castle	30.84
Edison	14.29
Red Bank	11.38
Slocum	9.26
Hidden Dome	2.81
Toborg	8.58
Brea	9.9
Shannon	11.67
Robinson	28.03
El Dorado	42.56
Shiells Canyon	13.76
Teapot Dome	9.24
Rock Creek	45.89
Plum Bush	33.46

**Table 4:** Average relative error (%) between simulated results obtained by different methods and experimental data.

Method	Training data	Test data
ANFIS	2.01	6.12
ANN	4.63	6.27
EOS	18.62	10.2

## Nomenclature

*a*: Pure component energy parameter
*a*_*i*_: Component *i* energy parameter
*a*_*ij*_: Composition and temperature dependent binary energy parameter
*a*_*ij*_^0^: Binary temperature dependent energy parameter
*A*_*m*_: Mixture temperature dependent term
*b*: Pure component volume parameter
*b*_*i*_: Volume parameter for component *i*
*B*: Dimensionless mixture parameter
*B*_*m*_: Mixture volume parameter
*d*_*ij*_: Binary interaction parameter for components *i* and *j*
*k*_*ij*_: Binary interaction parameter for components *i* and *j*
*P*: Pressure
*R*: Gas constant
*T*: Temperature
*V*: Mixture molar volume
*x*_*i*_: Mole fraction of component *i*
*Z*: Compressibility factor
*α*: Pure component temperature dependent term
*α*_*i*_: Temperature dependent term for component *i*.
